# Reply to the letter from Kumar et al.: Maastricht experience with the second-generation endoscopic laser balloon ablation system for the atrial fibrillation

**DOI:** 10.1007/s12471-015-0726-1

**Published:** 2015-07-02

**Authors:** P. Gal, A. Elvan

**Affiliations:** Cardiology Department of the Isala, Isala Klinieken, Dr. Van Heesweg 2, 8025 AB Zwolle, The Netherlands

The data reported in our manuscript [1] on the results on the endoscopically assisted system (EAS) in the ablation of atrial fibrillation (AF) are remarkably similar to the data reported by Kumar et al. [2] and in the current literature. Procedure duration is around 180 min, with a fluoroscopy time of around 30 min, although there appears to be a significant learning curve [3]. Furthermore, the EAS features a favourable safety profile, which was also observed in the analysis by Kumar.

There appears to be a difference in success after about 18 months follow-up between the two papers, namely 58 % in our study population, and 81 % in the population studied by Kumar. However, the difference is not statistically significant (chi square, *p*  = 0.074), which is probably caused by the limited sample size in both our study group (*n*  = 50) and Kumar’s (*n*  = 24). Moreover, there appear to be more patients in persistent AF in our analysis compared with Kumar’s (18 vs. 10 %) [4]. Of note, the success rate in our study is in line with previously published larger studies. Dukkipati et al. reported a 58.5 % success rate in 200 patients after a single EAS ablation [5].

At Heart Rhythm 2014, the results of the ADVICE (adenosine following pulmonary vein isolation to target dormant conduction elimination) trial were presented. This study showed that, in case of dormant conduction during adenosine administration, additional ablations targeting the ablation gaps resulted in an increased arrhythmia-free survival post-ablation. Although not yet widely applied, adenosine testing after pulmonary vein isolation to check for dormant conduction has already been demonstrated to result in higher ablation success rates, and Kumar et al. [4] should be credited for their excellent work in this field.

More recently, we reported the impact of pulmonary vein orientation on EAS ablation outcome [6]. Interestingly, arrhythmia-free survival varied between 21 and 88 % depending on left upper Pulmonary vein (PV) orientation. Furthermore, arrhythmia-free survival varied between 21 and 86 % depending on left lower PV orientation and arrhythmia-free survival varied between 29 and 88 % depending on right lower PV orientation. However, no association was found between right upper PV orientation and AF-free survival after EAS PV isolation.
